# Supplementary material for: Genome-wide CRISPR Screens in T Helper Cells Reveal Pervasive Crosstalk between Activation and Differentiation
Source: Cell. 2019 Feb 7;176(4):882–896.e18. doi: 10.1016/j.cell.2018.11.044 (PMC6370901; doi:10.1016/j.cell.2018.11.044)
Supplement: Data S2. Processed Data from All the Steps of the Analysis, Related to Figure 1 [file mmc2.zip › supplemental data/motif analysis/Pparg_homer/homerResults/motif20.similar.html]

motif20

## Information for motif20

A
C
T
G
A
C
G
T
C
G
T
A
A
C
G
T
C
G
T
A
A
C
G
T
C
G
T
A
C
G
T
A
A
G
T
C
A
C
G
T
  
Reverse Opposite:  

C
G
T
A
A
C
T
G
A
C
G
T
C
G
A
T
C
G
T
A
A
C
G
T
C
G
T
A
A
C
G
T
C
G
T
A
A
G
T
C
  

|  |  |
| --- | --- |
| p-value: | 1e-5 |
| log p-value: | -1.319e+01 |
| Information Content per bp: | 1.980 |
| Number of Target Sequences with motif | 13.0 |
| Percentage of Target Sequences with motif | 0.10% |
| Number of Background Sequences with motif | 7.7 |
| Percentage of Background Sequences with motif | 0.02% |
| Average Position of motif in Targets | 103.1 +/- 59.9bp |
| Average Position of motif in Background | 103.1 +/- 58.0bp |
| Strand Bias (log2 ratio + to - strand density) | 1.6 |
| Multiplicity (# of sites on avg that occur together) | 1.33 |
| Motif File: | file (matrix) reverse opposite |

### Similar de novo motifs found

|  |  |  |  |  |  |  |  |
| --- | --- | --- | --- | --- | --- | --- | --- |
| Rank | Match Score | Redundant Motif | P-value | log P-value | % of Targets | % of Background | Motif file |
